# Modulation of Gr1^low^ monocyte subset impacts insulin sensitivity and weight gain upon high-fat diet in female mice

**DOI:** 10.1038/ijo.2017.179

**Published:** 2017-08-29

**Authors:** S Béliard, W Le Goff, F Saint-Charles, L Poupel, V Deswaerte, L Bouchareychas, T Huby, P Lesnik

**Affiliations:** 1 INSERM, UMR_S U1166, Integrative Biology of Atherosclerosis Team, Paris, France; 2 APHM, La Conception Hospital, Endocrinology, Nutrition and Metabolic Diseases Department, Marseille, France; 3 AMU, Marseille, France; 4 Sorbonne Universite Paris 06, UMR_S1166, Paris, France; 5 Institute of Cardiometabolism and Nutrition (ICAN), Groupe Hospitalier Pitié-Salpétrière, Paris, France

## Abstract

**Background/Objectives:**

Blood monocytes are expanded during obesity. However, the differential contribution of monocyte subsets in obesity-related metabolic disorders remains unknown. The aim of the study was to define the role of the Gr1^low^ monocyte subset upon high-fat diet (HFD).

**Methods:**

We used transgenic female mouse models allowing the modulation of circulating Gr1^low^ monocyte number (decreased number in CX3CR1^−/−^ mice and increased number in CD11c-hBcl2 mice) and studied obesity upon HFD.

**Results:**

We reported here that HFD induced monocytosis in mice, preferentially due to Gr1^low^ monocyte expansion, and was associated with a specific upregulation of CD11c on that subset. Using mice models with altered Gr1^low^ monocyte number, we found a striking correlation between Gr1^low^ monocytes, bodyweight (BW) and insulin resistance (RT) status. Indeed, CX3CR1^−/−^ female mice, with reduced Gr1^low^ monocytes upon HFD, showed increased RT and a pro-inflammatory profile of the adipose tissue (AT) despite a lower BW. Conversely, mice expressing the anti-apoptotic gene *hBcl2* in CD11c-expressing cells have increased Gr1^low^ monocytes, higher insulin sensitivity upon HFD and an anti-inflammatory profile of the AT. Finally, increasing Gr1^low^ monocytes in Gr1^low^-defective CX3CR1^−/−^ mice rescued BW loss in these mice.

**Conclusions:**

By using transgenic female mice and adoptive transfer experiments, we established the evidence for a correlation between Gr1^low^ monocyte subset and weight gain and RT. Hence, this specific Gr1^low^ monocyte subset could be used as a target for acting on AT inflammation and RT.

## Introduction

Obesity and its associated complications such as type 2 diabetes, cardiovascular diseases, cancers and premature mortality has become a worldwide health problem. Over the last decade, growing evidences have emerged bringing up a close link between obesity and immunity.^1^ Obesity is characterized by a chronic low-grade inflammation generating metabolic complications.^2^ This low-grade inflammation occurred at a systemic level as well as in different tissues. Indeed, in adipose tissue (AT), in liver and muscles, obesity-induced inflammation is characterized by an infiltration of pro-inflammatory macrophages (called M1) secreting pro-inflammatory cytokines such as tumor necrosis factor-α and interleukin-6.^3^ These pro-inflammatory cytokines are thought to be responsible for insulin resistance (IR) locally and at a systemic level.^4, 5^ On the other hand, in lean subjects, alternatively activated macrophages (called M2) are important for maintaining homeostasis in AT.^6^

Blood monocytes, the precursors of macrophages, have a pivotal role in innate immunity.^7^ They are key actors in septic and aseptic inflammation diseases. Monocytes are divided into at least three functional subsets in humans and two functional subsets in mice. These subsets differ in the cytokines, chemokine receptors and adhesion molecules they produce and in their functions.^8, 9^ In mice, one subset of monocytes, characterized by their surface expression of Gr1^high^, CCR2 and a low level of CX3CR1, are selectively recruited in inflammatory sites. They are called ‘inflammatory’ or ‘classical’ monocytes. The other subset ‘Gr1^low^ monocytes’ (non-classical monocytes), which is characterized by a reciprocal marker expression profile (CX3CR1^high^ and CCR2−) was initially called ‘resident monocytes’ due to their ability to replenish tissues in resting conditions after adoptive transfer.^8^ Literature to date suggests that Gr1^low^ monocytes are recruited to inflammatory sites^10, 11, 12^ or alternatively originates from a phenotypic switch of Gr1^high^ monocytes in inflammatory tissues.^13, 14, 15^ In addition, recent data demonstrated that this specific subset of monocytes is an ‘intravascular housekeeper’ as Gr1^low^ monocytes orchestrate the phagocytosis of damaged endothelial cells by polynuclear neutrophils and contribute to the resolution of inflammation.^16^

Inflammation is clearly associated to the pathophysiology of obesity and IR. However, the relative contribution of the different subsets of monocytes in the development of AT inflammation is still poorly understood. In human, clinical studies have revealed that an increase of a specific subset of monocytes was associated to obesity.^17, 18^ An expansion of the CD14^dim^CD16^+^ monocyte subset in human, corresponding to Gr1^low^ monocyte subset in mice,^19^ was found to be correlated with increased fat mass and glycemia.^17^ Similarly, in the present study, we found that diet-induced obesity (DIO) promotes a large expansion of the specific Gr1^low^ monocyte subset in mice. In addition DIO increases, specifically on the Gr1^low^ subset, the expression of CD11c, a major β-2 integrin involved in adhesion during rolling of monocytes on endothelial cells. Thus, to gain more insight into the role of Gr1^low^ monocytes during obesity, we used female mice models, which are associated with major changes in the proportion of Gr1^low^ monocytes in blood: CX3CR1^−/−^ mice; CD11c-hBcl2 mice; and the double genetically modified CX3CR1^−/−^/CD11c-hBcl2 mice. Indeed, CX3CR1 is a chemokine receptor highly expressed on Gr1^low^ monocytes and a key factor for Gr1^low^ survival.^20^ CX3CR1 deficiency in mice results in a strong decrease in Gr1^low^ monocytes population.^20, 21^ By contrast, we used a transgenic model (that is, CD11c-hBcl2 mice) displaying higher levels of Gr1^low^ monocytes in blood. CD11c-hBcl2 mice model takes advantage of the expression of an anti-apoptotic gene (*Bcl-2*) under the control of the CD11c promoter thus enhancing specifically cell survival of CD11c-positive cells.^22^ By using these mouse models, we found a striking correlation between the level of circulating Gr1^low^ monocytes and the bodyweight (BW). We also provide data supporting a new key protective role for Gr1^low^ monocytes against IR associated with obesity.

## Materials and methods

### Experimental animals

Littermate female CX3CR1−/− (ref. 23) and wild-type (WT) controls on a C57BL/6 background were fed either a normal chow diet or a high-fat diet (HFD) for 30 weeks or indicated time. For bone marrow (BM) transplantation, female mice C57BL/6 were irradiated and transplanted with donor’s BM: CX3CR1^−/−^; CD11c-hBcl2;^24^ CD11c-hBcl2/CX3CR1^−/−^; or C57BL/6 mice, as previously described.^25^ All animal procedures were performed with accreditation from the French government and under strict compliance with Animal Welfare Regulations. Blood collection for monocyte study was done in the morning between 0800 and 1000 hours.

### Flow cytometry

For analyze of AT stromal vascular fraction (SVF), peri-ovarian fat pads were excised and minced in phosphate-buffered saline. Tissue suspension was incubated with collagenase D (Roche, Bâle, Suisse) at 2.5 mg ml^−1^ at 37 °C for 1 h with shaking. After digestion, AT cell suspensions were spun at 200 *g* for 10 min to separate floating adipocytes to SVF pellet. The cell suspension was filtered through a 100 μm filter and then spun at 700 *g* for 5 min. SVF cell preparation, as hematopoietic cell preparation, was done as previously described.^25^ Antibodies are described in Supplementary Table 1. Data were acquired on LSRFortessa cell analyzer (Becton Dickinson, Franklin Lakes, NJ, USA) and analyzed with FACSDiva Software (Becton Dickinson Biosciences, Franklin Lakes, NJ, USA). Gating strategy for isolating the populations of Gr1^low^ and Gr1^high^ monocytes is shown in Supplementary Figure 6.

### Metabolic evaluation

Glucose tolerance test (GTT) and insulin tolerance test (ITT) were performed after overnight fast with, respectively, intraperitoneal injection of 1 mg g^−1^ body dextrose or intraperitoneal injection 1.5 mUI g^−1^ body insulin (Actrapid, Novo Nordisk, Bagsværd, Danemark). Plasma insulin levels were evaluated with Ultrasensitive mouse Insulin ELISA (Mercodia France, Paris, France). Plasma lipid analyses were done as previously described.^25^

### Analysis of adipose tissue gene expression by quantitative PCR

RNA extraction from 100 mg sample of peri-ovarian adipose tissue was performed with a RNeasy Lipid Tissue Mini kit (QIAGEN, Venlo, Netherlands). RNA preparation and quantitative PCR were done as previously described.^25^

### Statistical analysis

The statistical significance of the differences between groups was evaluated using the Mann–Whitney *U*-test for unpaired comparisons. The linear correlation between Gr1^low^ circulating monocytes and BW or homeostasis model accessment of insuline resistance (HOMA Index) was mesured by the Pearson’s coefficient. Comparisons between three groups were analyzed using repeated-measures two-way analysis of variance.

## Results

### HFD induces a marked increase of the Gr1^low^ monocyte subset

**Figure 1:**
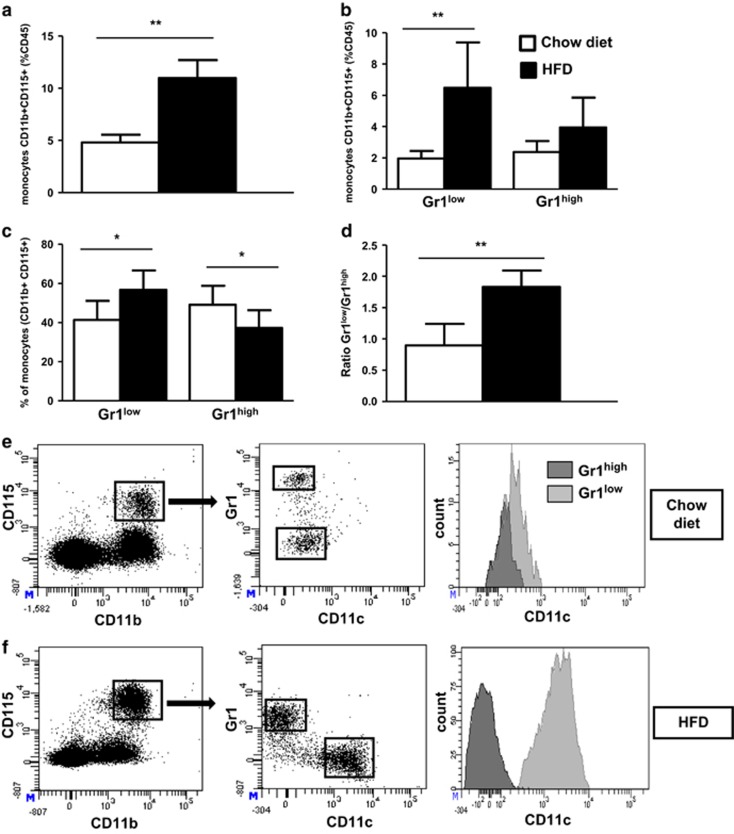
Gr1^low^ monocytes are increased in DIO, and the integrin CD11c is highly upregulated on Gr1^low^ monocytes during HFD in C57BL/6 mice. (**a**) The frequency of monocytes was determined by flow cytometry in C57BL/6 female mice fed a chow diet (white histograms) or a HFD containing 60% kcal (black histograms) from fat for 30 weeks, based on expression of CD45, CD11b and CD115 (respectively, *n*=6 and 12). (**b**, **c**) Relative frequencies of Gr1^low^ and Gr1^high^ subsets, in percentage of leucocytes (CD45^+^) (**b**) or of monocytes (**c**). (**d**) Ratio of Gr1^low^/Gr1^high^ monocytes. (**e**, **f**) Dot plots of the CD11c expression on Gr1^low^ and Gr1^high^ monocytes (**e**: chow diet; **f**: HFD). *P<0.05. **P<0.01. Data are expressed as means±s.d.

Previous studies have documented monocytosis in mice fed a HFD.^26, 27^ To assess the different populations of monocytes during DIO, we analyzed changes in white blood cell populations in C57BL/6 mice fed a standard chow diet or a HFD. The percentage of blood monocytes was more than doubled in mice maintained on HFD for 30 weeks (Figure 1a). This monocytosis was mainly due to a threefold increase of Gr1^low^ population (Figure 1b) leading to a twofold increase in the Gr1^low^/Gr1^high^ ratio (0.9±0.3 on chow diet vs 1.8±0.7 on HFD, *P*=0.008; Figures 1c and d). Wu *et al.*^27^ reported in both human and mice that obesity was associated with a strong expression of the integrin CD11c on monocytes. However, the differential repartition of this integrin between the two subsets of monocytes upon HFD has not been studied yet. Here we observed that Gr1^low^ monocytes expressed higher levels of CD11c compared with Gr1^high^ monocyte subset in mice maintained on a chow diet (358±130 vs 186±71 mean fluorescence intensity, *P*<0.001; Figure 1e). Furthermore, the expression levels of CD11c were strongly upregulated specifically on Gr1^low^ monocyte subset upon HFD (904±145 mean fluorescence intensity on Gr1^low^ subset vs 178±124 mean fluorescence intensity on Gr1^high^ subset, *P*<0.001; Figure 1f).

### CX3CR1^−/−^ female mice fed a HFD display a decrease of Gr1^low^ monocyte subset associated with an increased RT

**Figure 2:**
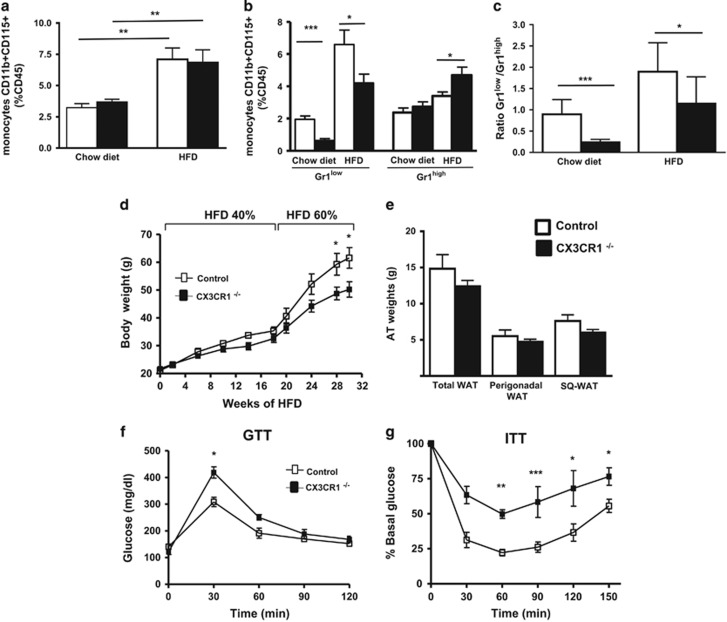
Gr1^low^ monocytes are decreased in CX3CR1^−/−^ HFD-fed mice, and CX3CR1 deficiency results in decreased BW gain but increased IR following exposure to HFD. (**a**) The frequency of monocytes was determined in C57BL/6 or CX3CR1^−/−^ female mice fed a chow diet (white histograms) or a HFD containing 60% kcal from fat (black histograms) for 30 weeks, as in Figure 1 (respectively, *n*=9 and 12). (**b**) Relative frequencies of Gr1^low^ and Gr1^high^ monocytes, in percentage of leucocytes (CD45^+^). (**c**) Ratio of Gr1^low^/Gr1^high^ monocytes. (**d**) Weight gain in CX3CR1^−/−^ mice and control. Mice were fed a diet of 40% kcal from fat until 18 weeks then shifted to a diet of 60% kcal from fat (*n*=6 controls and 10 CX3CR1^−/−^ mice). (**e**) AT weights in CX3CR1^−/−^ and control mice fed 30 weeks with the HFD described in **e**. (**f**, **g**) Plasma glucose during GTT (**f**) and ITT (**g**) after 12 h fasting after 28 weeks of HFD for GTT and 30 weeks of HFD for ITT (*n*=5/4; HFD containing 40% followed by 60% kcal from fat). *P<0.05. **P<0.01. ***P<0.001. Data are expressed as means±s.d.

Landsman *et al.*^20^ have shown that CX3CR1–CX3CL1 interaction is critically involved in cell survival of the Gr1^low^ subset. Thus, we hypothesized that CX3CR1 deficiency would counterbalance the rise in monocytosis of Gr1^low^ monocytes associated with HFD. Therefore, we evaluated blood monocyte subsets in CX3CR1^−/−^ female mice fed with HFD (Figure 2). The percentage of blood monocytes was unchanged in CX3CR1^−/−^ mice as compared to control mice maintained either on a chow diet or on a HFD (Figure 2a). As previously described, Gr1^low^ monocyte population was markedly reduced (−68%) in CX3CR1^−/−^ mice fed a chow diet^20, 21^ compared to controls (Figures 2b and c). Similarly, in CX3CR1^−/−^ mice under HFD conditions, we showed that Gr1^low^ monocytes were also significantly reduced (−35%) (Figure 2b). The Gr1^low^/Gr1^high^ ratio was found to be decreased nearly by twofold in CX3CR1^−/−^ mice fed a HFD as compared to control mice (respectively, 1.1±0.5 and 1.9±0.7, *P*=0.02; Figure 2c). Results on the circulating monocytes were identical at 22 and 30 weeks of HFD (only data with HFD containing 60% kcal from fat are shown in the Figure 2).

As inflammation has been associated with metabolic disorders, we hypothesized that an imbalance in Gr1^low^ population may influence the metabolic phenotype of mice fed a HFD. To test this, CX3CR1^−/−^ and control mice were fed a HFD containing 40% of kcal from fat for 18 weeks. This resulted in a slightly lower BW in CX3CR1^−/−^ mice as compared to controls (−8%, *P*=0.19; Figure 2d). These mice were further challenged with a fatter HFD containing 60% of kcal from fat for 12 more weeks. Then, CX3CR1^−/−^ mice showed a significant lower BW gain as compared to WT controls (Figure 2d). CX3CR1^−/−^ mice had a trend to reduce total AT mass due to a reduction of perigonadal fat and subcutaneous fat (Figure 2e). We measured adipocyte size after 30 weeks of HFD. As expected for leaner mice, we observed that adipocyte size in the perigonadal AT of CX3CR1^−/−^ mice was significantly decreased compared to control mice (Supplementary Figure 1a). Surprisingly, despite a lower BW, GTT, oral GTT and ITT evaluated in BW-matched mice upon DIO demonstrated that CX3CR1^−/−^ mice were significantly more insulin-resistant than WT controls (Figures 2f and g; Supplementary Figure 1b).

### Hematopoietic cells are responsible for the lower BW gain and the increased IR observed in CX3CR1 deficiency female mice upon HFD

**Figure 3:**
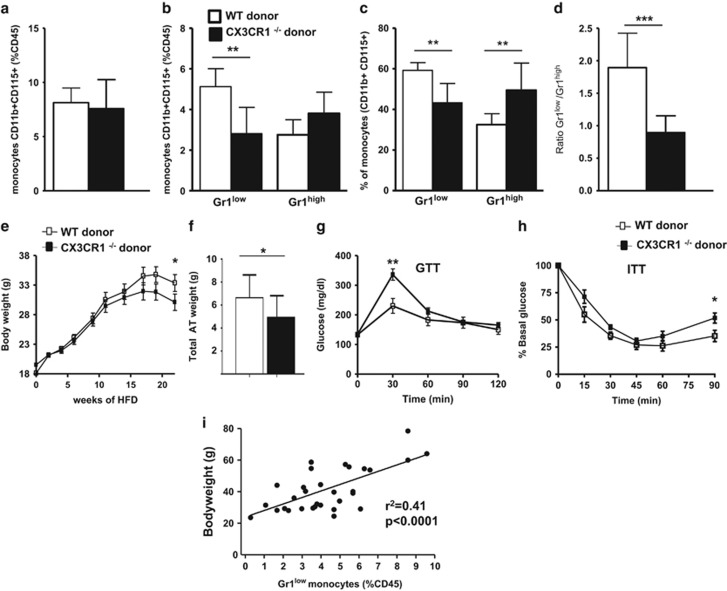
Mice deficient for CX3CR1 in hematopoietic cells have the same phenotype as mice totally deficient for CX3CR1 upon chronic HFD. (**a**) The % of monocytes was determined as described in Figure 1 of CX3CR1^−/−^ donor mice (black histograms; *n*=11) and WT donor mice (white histograms; *n*=12) fed a HFD containing 60% kcal from fat for 22 weeks. (**b–d**) Relative frequencies of Gr1^low^ and Gr1^high^ monocytes, in percentage of leucocytes (CD45^+^) (**b**) or of monocytes (**c**), and the ratio of Gr1^low^/Gr1^high^ monocytes (**d**) are shown. (**e**) Weight gain and total AT weight (**f**) in CX3CR1^−/−^ donor mice (*n*=11) and WT donor mice (*n*=12) fed a diet of 60% kcal. Plasma glucose during GTT (**g**) and ITT (**h**) after 12 h fasting at 24 and 26 weeks HFD 60% (*n*=8 CX3CR1^−/−^ donor and 7 WT donor matched to the weight). (**i**) Correlation between BW and the rate of circulating Gr1^low^ monocytes (%CD45) in mice deficient for CX3CR1 (CX3CR1^−/−^ and recipients of CX3CR1^−/−^ BM) and their controls (respectively, C57Bl/6 and controls BM) fed a HFD (*n*=32 mice). *P<0.05. **P<0.01. ***P<0.001. Data are expressed as means±s.d.

To confirm that the metabolic phenotype observed in CX3CR1^−/−^ mice fed a HFD was supported by the hematopoietic system, we generated BM chimeras with BM of C57BL/6 or CX3CR1^−/−^ female mice fed with normal chow diet. After 22 weeks of HFD containing 60% of kcal from fat, the monocytosis was unchanged between mice receiving CX3CR1^−/−^ and C57BL/6 donor cells (Figure 3a). Upon HFD, a significant decrease of the Gr1^low^ monocyte subset was observed in mice deficient for CX3CR1 in the hematopoietic system (−41%, *P*=0.001; Figure 3b), which was closed to that observed in mice totally deficient for CX3CR1 (−35%, *P*=0.02; Figure 2b), when compared with the respective HFD-fed control mice. The Gr1^low^/Gr1^high^ monocyte ratio was decreased by twofold in CX3CR1^−/−^ BM chimeric mice as compared with controls (Figure 3d). Further evaluation of the metabolic phenotype revealed lower BW in CX3CR1^−/−^ BM chimeric mice than controls (Figure 3e). CX3CR1^−/−^ BM chimeric mice had a decrease of total fat mass (−25.6%, *P*=0.05; Figure 3f). In agreement with the phenotype observed in mice totally deficient for CX3CR1, and despite a lower BW, CX3CR1^−/−^ BM chimeric mice displayed increased IR as assessed by GTT and ITT (Figures 3g and h). Consistently, circulating non-esterified fatty acids levels were significantly increased in CX3CR1^−/−^ BM chimeric mice as compared to controls (respectively, 1.56±0.08 vs 1.09±0.04 mg dl^−1^, *P*<0.0001). Remarkably, when we pooled all mice deficient for CX3CR1 (totally deficient and deficient in the hematopoietic compartment) and their controls, we found a positive correlation between levels of circulating Gr1^low^ monocytes and BW (Figure 3i). Each correlation was also significant for each experimental group evaluated separately (Supplementary Figure 2). By contrast, we did not find any correlation between the rate of Gr1^high^ monocytes and the BW (Supplementary Figure 3).

### CX3CR1 deficiency exacerbates HFD-induced inflammation within AT

**Figure 4:**
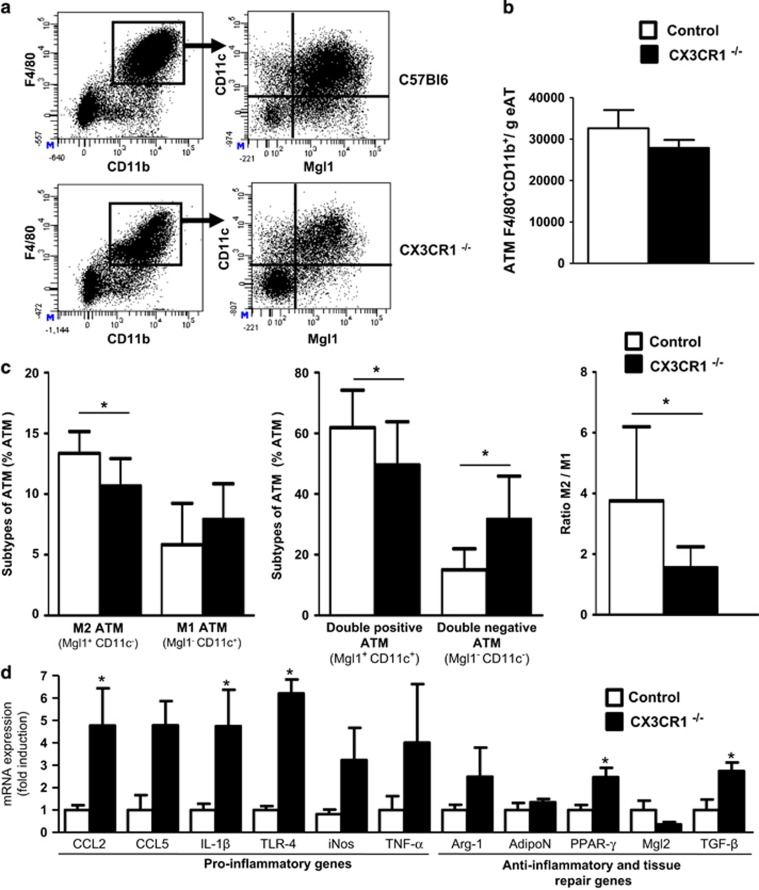
Characterization of AT macrophage in CX3CR1^−/−^ fed a HFD. (**a**) Dot plots of macrophages (F4/80^+^CD11b^+^CD45^+^ cells) in AT in CX3CR1^−/−^ mice and control mice fed a diet of 60% kcal from fat for 30 weeks. (**b**) Histograms represent ATM content assessed by flow cytometry in CX3CR1^−/−^ mice (*n*=9) and WT controls (*n*=8) treated as in **a**. (**c**) Characterization of M2 and M1 macrophages based on the expression of CD11c and Mgl1. Characterization of double-positive and double-negative macrophages for the two markers CD11c and Mgl1. Ratio of M2/M1 macrophages in mice described in **b**. (**d**) Levels of mRNA expression of functional M1 and M2 markers were evaluated by qPCR in adipose tissue from mice described in **a** (*n*=4 controls and 4 CX3CR1^−/−^ mice, representative of two independent experiments). Relative mRNA expression of M1 and M2 genes was normalized to the average expression of two housekeeping genes (hypoxanthine guanine phosphoribosyl transferase and ribosomal protein S3). *P<0.05. Data are expressed as means±s.d.

During obesity, monocytes are recruited in AT^28^ and differentiate into AT macrophages (ATMs). We analyzed the different populations of ATM by flow cytometry using markers of pro-inflammatory M1 macrophages (CD45^+^F4/80^+^CD11c^+^Mgl1^neg^) and M2 anti-inflammatory and repairing macrophages (CD45^+^F4/80^+^Mgl1^+^CD11c^neg^; Figure 4a). The number of ATM per gram of perigonadal fat was similar in both CX3CR1^−/−^ mice and controls (Figure 4b). However, the distribution of each subset of ATM based on the membrane expression of Mgl1 and CD11c was altered in CX3CR1^−/−^ mice (Figure 4c). Indeed, in CX3CR1^−/−^ mice, the % of M2 was significantly diminished (10.7±2.2% M2 in CX3CR1^−/−^ mice vs 13.3±1.8% M2 in controls, *P*=0.04) when the % of M1 macrophages was not significantly increased (7.4±2.8% M1 in CX3CR1^−/−^ mice vs 5.9±3.2% M1 in controls, *P*=0.4). These changes in ATM population resulted in reduction by more than a half of the M2/M1 ratio in CX3CR1^−/−^ mice as compared to controls (respectively, 1.5±0.7 vs. 3.8±2.4, *P*=0.03; Figure 4c). The two other analyzed population of ATM (double-positive or double-negative for Mgl1 and CD11c) were also significantly modified in CX3CR1^−/−^ mice compared to controls (Figure 4c). However, the origin and the precise role of these two populations are poorly known, even though it seems that the double-positive population, which expresses a mixed M1/M2 transcriptional profile tends to favor a M2 phenotype with chronic HFD.^29^ We also observed that this double-positive population was significantly decreased in CX3CR1^−/−^ mice as compared to controls (49.5±14.3% in CX3CR1^−/−^ mice vs 61.9±12.4% in controls, *P*=0.04).

We then examined inflammatory gene expressions in perigonadal AT of CX3CR1^−/−^ mice and controls (Figure 4d). We found an upregulation of pro-inflammatory genes in CX3CR1^−/−^ mice as compared to controls. In particular, chemokine ligand 2, interleukin-1β and Toll-like receptor-4 mRNA were significantly increased by about fourfold while mRNAs for genes associated with a M2 phenotype were not significantly changed.

### Expression of the anti-apoptotic gene *hBcl2* under the CD11c promoter increases Gr1^low^ monocyte levels in DIO conditions and promotes insulin sensitivity

**Figure 5:**
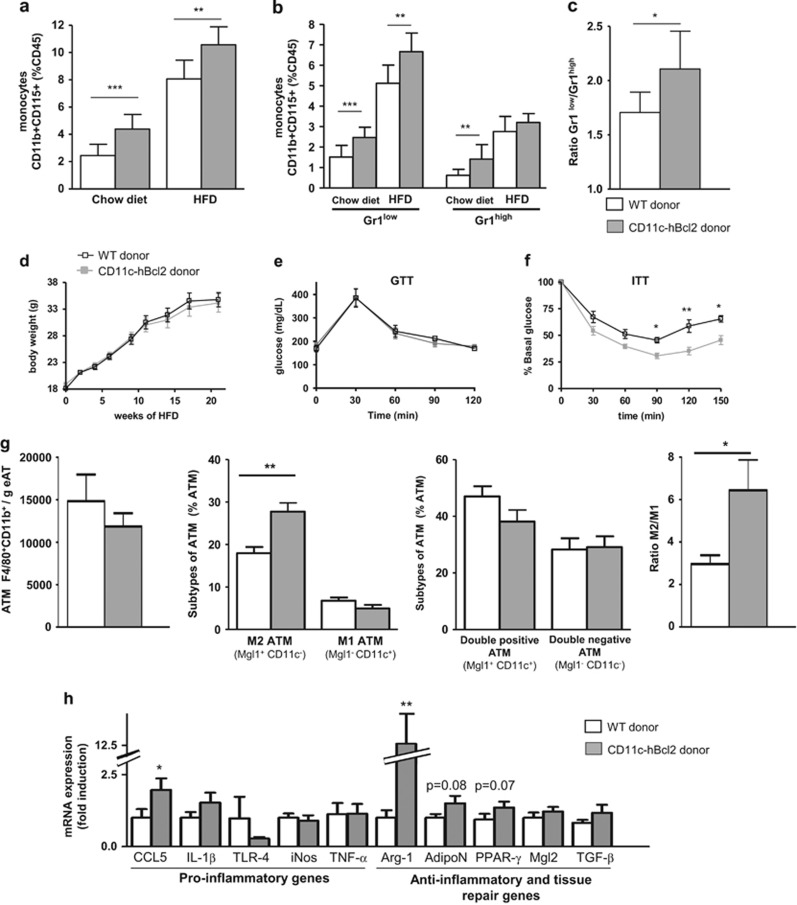
Bcl-2 expression in CD11c^+^ cells increases the Gr1^low^ monocyte proportion, insulin sensitivity and M2 ATM content in DIO conditions. (**a**) Blood monocyte levels characterized as in Figure 1 in irradiated C57Bl/6 mice recipients of WT (white histograms, *n*=8) or transgenic CD11c-hBcl2 BM cells (gray histograms, *n*=9) fed a HFD for 22 weeks. (**b**) Relative frequencies of Gr1^low^ and Gr1^high^ monocytes in mice described in **a**. (**c**) Histogram shows the ratio Gr1^low^/Gr1^high^ monocytes in mice described in **a–c**. (**d**) Weight gain in irradiated C57Bl/6 mice transferred with Tg CD11c-hBcl2 BM cells (gray) or with WT BM cells (white). Mice fed a diet of 60% kcal from fat (*n*=10 Tg CD11c-hBcl2 and 10 controls). Plasma glucose during GTT (**e**) and ITT (**f**) in mice described in **d** fed a HFD for 17 weeks and after 12 h fasting (*n*=6/6). (**g**) ATM content in mice described in **a**. Characterization of ATMs based on the expression of Mgl1 and CD11c. Ratio of M2/M1 macrophages. (**h**) Levels of mRNA expression of functional M1 and M2 markers were evaluated by quantitative PCR in AT from mice described in **a** (*n*=10 controls and 7 CD11c-hBcl2 transgenic mice). Relative mRNA expression of M1 and M2 genes was normalized to the average expression of two housekeeping genes (hypoxanthine guanine phosphoribosyl transferase and ribosomal protein S3). *P<0.05. **P<0.01. Data are expressed as means±s.d.

We further used a complementary mouse model in which the Gr1^low^ monocyte population in blood is increased. We generated BM chimeric mice with BM isolated from CD11c-hBcl2 mice or C57BL/6 mice. After 22 weeks of HFD containing 60% of kcal from fat, we observed that CD11c-hBcl2 BM chimeric mice displayed significantly increased monocyte levels as compared to controls both on normal chow diet and on HFD (Figure 5a). On chow diet, this monocytosis was due to an expansion of both populations of monocytes but was restricted solely to the Gr1^low^ population upon HFD reflecting expression levels of CD11c (Figure 5b). Accordingly, the Gr1^low^/Gr1^high^ ratio was significantly increased in CD11c-hBcl2 BM chimeric mice as compared to controls upon DIO conditions (Figure 5c).

We next investigated the metabolic status in CD11c-hBcl2 BM chimeric mice. No differences in the weight gain were found between CD11c-hBcl2 BM chimeric mice and controls over the time period (Figure 5d). However, ITT demonstrated that HFD-fed CD11c-hBcl2 BM chimeric mice were significantly more insulin sensitive than WT controls (Figure 5f). Consistently, circulating triglycerides and non-esterified fatty acids were significantly reduced in CD11c-hBcl2 BM chimeric mice compared to control (respectively, 115±5.7 vs 133±4.1 mg dl^−1^, *P*=0.02; and 0.91±0.07 vs 1.09±0.03 mg dl^−1^, *P*=0.04). Adipocyte size of recipient mice for CD11c-hBcl2 BM was smaller than those of recipient of WT BM (Supplementary Figure 4a), which was consistent with the increase insulin sensitivity we observed.

We then quantified the ATM content in perigonadal fat and found that the number of macrophages per gram of AT was not significantly modified in CD11c-hBcl2 BM chimeric mice as compared to controls (Figure 5g). However, the % of M2 macrophages was significantly increased in CD11c-hBcl2 BM chimeric mice compared to controls (respectively, 27.8±6.1% vs 18.0±4.9%, *P*=0.003). The percentage of M1 ATM and the percentage of double-positive cells (Mgl1^+^CD11c^+^) or double-negative cells (Mgl1^−^CD11c^−^) were not different between groups (Figure 5g). Finally, the M2/M1 ratio in AT was more than double in CD11c-hBcl2 BM chimeric mice compared to controls (respectively, 6.4±4.0 vs 3±1.4, *P*=0.02; Figure 5g). We then confirmed that anti-inflammatory and tissue repair genes were more expressed in CD11c-hBcl2 BM chimeric mice compared to controls (Figure 5h). In particular, Arginase-1 mRNA levels were found 12-fold more elevated in CD11c-hBcl2 BM chimeric mice than that in controls.

### Enforced expression of hBcl2 in Gr1^low^ monocytes partially rescues the Gr1^low^/Gr1^high^ ratio and fully rescues the weight loss in CX3CR1^−/−^ female mice upon HFD

**Figure 6:**
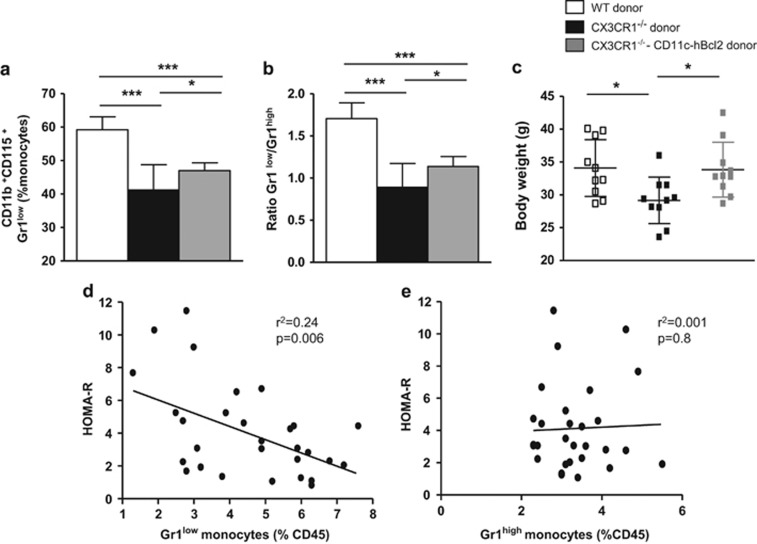
Expression of hBcl2 in Gr1^low^ monocytes partially rescue the ratio Gr1^low^/Gr1^high^ and totally rescue the weight loss in CX3CR1-deficient mice in HFD conditions. (**a**) Histogram shows the proportion of Gr1^low^ monocytes on total blood monocytes in irradiated C57Bl/6 mice transferred with WT BM cells (white) or CX3CR1^−/−^ BM cells (black) or CX3CR1^−/−^/CD11c-hBcl2 Tg BM cells (gray) fed a HFD for 22 weeks (60% kcal from fat; *n*=10 WT, 10 CX3CR1^−/−^ and 10 CX3CR1^−/−^/CD11c-hBcl2 Tg). (**b**) Histogram shows the ratio Gr1^low^/Gr1^high^ monocytes in mice described in **a**. (**c**) Weights at 22 weeks of HFD in mice treated as in **a**. (**d**) Correlation between IR evaluated with HOMA-R indices and the rate of Gr1^low^ monocytes in mice recipients transferred with WT BM cells or CX3CR1^−/−^ BM cells, or CD11c-hBcl2 Tg BM cells or CX3CR1^−/−^/CD11c-hBcl2 Tg BM cells and fed a HFD for 22 weeks (*n*=8 C57Bl/6, *n*=8 CX3CR1^−/−^, *n*=8 CD11c-hBcl2, *n*=8 CX3CR1^−/−^/CD11c-hBcl2 mice. (**e**) Correlation between HOMA-R indices and the rate of circulating Gr1^high^ monocytes in the same mice as in **d**. *P<0.05. **P<0.01. ***P<0.001. Data are expressed as means±s.d.

Given the opposite phenotype observed in CX3CR1^−/−^ mice and in CD11c-hBcl2 transgenic mice, we hypothesized that the expression of the anti-apoptotic protein Bcl-2 under the control of CD11c in CX3CR1^−/−^ mice might rescue the proportion of Gr1^low^ monocytes, the Gr1^low^/Gr1^high^ ratio and as a consequence the metabolic phenotype in DIO conditions. To test this, we generated BM chimeric mice with BM cells isolated from three groups of donor mice: (i) CX3CR1^−/−^; (ii) CD11c-hBcl2/CX3CR1^−/−^; and (iii) C57BL/6. After 22 weeks on HFD, percentage of Gr1^low^ monocytes was significantly increased in CD11c-hBcl2/CX3CR1^−/−^ BM chimeric mice compared to CX3CR1^−/−^ BM chimeric mice (Figure 6a). However, these Gr1^low^ monocyte levels did not reach those of C57BL/6 BM chimeric mice. Accordingly, we observed that the Gr1^low^/Gr1^high^ ratio of CD11c-hBcl2/CX3CR1^−/−^ BM chimeric mice was slightly but significantly increased as compared to CX3CR1^−/−^ BM chimeric mice (Figure 6b). Hence, the expression of the anti-apoptotic *hBcl2* gene under the control of CD11c promoter partially rescued the decrease in percentage of Gr1^low^ monocytes in blood of CX3CR1^−/−^ mice. Upon HFD, the weight gain of CD11c-hBcl2/CX3CR1^−/−^ BM chimeric mice was similar to the one observed in control mice (Figure 6c). These results showed that expression of hBcl2 under the control of the CD11c promoter totally rescued the phenotype of lower BW gain in CX3CR1^−/−^ mice upon HFD. Moreover, if we considered all different mouse models used in this study, we found a strong inverse correlation between the % of Gr1^low^ monocytes and the IR evaluated with the HOMA index (Figure 6d). No correlations were found between the rate of Gr1^high^ monocytes and the HOMA index in the same mouse models (Figure 6e).

## Discussion

The differential contributions of monocyte subsets to the pathophysiology of metabolic diseases is controversial and not fully defined. Using transgenic mouse models, which differ in the proportion of circulating Gr1^low^ monocytes under HFD, we established a strong correlation between the number of circulating Gr1^low^ monocytes and BW gain and insulin sensitivity. We demonstrated that chronic HFD leads to a specific expansion of Gr1^low^ monocyte subset and induces an increased surface expression of the integrin CD11c specifically on Gr1^low^ monocytes. Moreover, our data indicate that expansion of Gr1^low^ monocytes is correlated with diminished HFD-induced inflammation associated to a favorable M2/M1 ratio in the AT. All these results are in favor of a protective role of Gr1^low^ monocytes during obesity.

In the present study, we have shown that HFD was associated with expansion of both monocyte subsets, but of greater magnitude for the Gr1^low^ subset (threefold increase). This result is in accordance with the study of Morris *et al.*^30^ who observed a monocytosis predominantly associated with the Gr1^low^ subset in mice fed a HFD for 40 weeks while other studies reported a HFD-induced monocytosis, which were not selectively associated with a Gr1^low^ or Gr1^high^ subsets.^26, 27, 28, 29, 30, 31^ Our results are consistent with studies in human, showing that monocyte count is positively associated with body mass index^18^ and that higher levels of ‘non-classical’ monocyte subset (corresponding to GR1^low^ subset in mice) are associated with increased body fat mass, as well as with glycemia.^17, 18^ A recent work confirmed that the number of ‘non-classical’ subset of monocytes was increase in subjects with metabolic syndrome compared to control subjects.^32^

This study stresses a role for Gr1^low^ monocytes in weight gain during HFD feeding. The transfer of CX3CR1^−/−^ BM in WT irradiated female mice experiments clearly demonstrated that a hematopoietic defect influences weight gain during HFD. Moreover, the striking correlation we put forward between the circulating Gr1^low^ monocytes and the BW is in favor of a possible role for Gr1^low^ monocytes in weight gain and AT expansion. Consistently, the CD11c-hBcl2-driven Gr1^low^ monocyte subset rescue in CX3CR1^−/−^ background totally restores the BW, although CD11c-hBcl2 expression in WT mice has no impact on this parameter. The mechanistic role involving blood monocytes in weight gain is still poorly understood and necessitates further studies.

The IR observed in CX3CR1^−/−^ female mice under HFD is of particular interest. Here we demonstrate that CX3CR1 deficiency led to an increased inflammation of AT associated to a significantly decrease by more than twofold of the M2/M1 ratio. Consistent with our hypothesis that circulating Gr1^low^ monocytes are protective, reverse strategy using CD11c-hBcl2 transgenic mice showed that increased circulating Gr1^low^ subset is associated with an increased M2 ATM population, an increased tissue repair function and higher insulin sensitivity. In a consistent manner, in mouse lacking Gr1^low^ monocytes (for example, deficient in differentiation factor Nur4a)^33^ lower levels of Gr1^low^ monocytes was associated with increased IR under HFD.^34, 35^ However, the mechanism linking circulating Gr1^low^ monocytes and AT functionality is unknown. It was first hypothesized that M2 macrophages are derived from the pool of circulating Gr1^low^ monocytes. In an experimental model of heart infarction, Nahrendorf *et al.*^12^ first proposed a biphasic model of macrophage accumulation (that is, M1 macrophages derived from Gr1^high^ monocytes, and M2 macrophages derived from Gr1^low^ monocytes) characterized by sequential recruitment of Gr1^low^ monocytes in a CX3CR1-dependent manner and of Gr1^high^ monocytes in a CCR2-dependent manner. Recently, by combining advanced flow cytometry and fate-mapping approaches, the same group revisited the model and observed that M2 macrophages accumulated in the reparative phase were derived from Gr1^high^ monocytes during myocardial infarction.^14^ Our findings are consistent with those of Satoh *et al.*^36^ who used mice depleted in M2 macrophages in AT (Trib1^−/−^ mice) and demonstrated that the M2 ATM deficiency was responsible for an increase IR, an increase AT inflammation and, very interestingly, a reduction in fat mass, similar to our findings in CX3CR1^−/−^ mouse female model. BM transfer studies further confirm that the effects of Trib1 deficiency on AT were supported by the hematopoietic cells.^36^ Another hypothesis linking Gr1^low^ monocytes and AT functions is link to their primary function of patrolling the vascular endothelium and monitoring its integrity. Such role would afford protection of AT vessels and protect the AT from inflammation. Indeed, Carlin *et al.*^16^ demonstrated that in response to a ‘danger signal’ expressed by the renal endothelium, Gr1^low^ monocytes were intravascular retained, allowing neutrophils recruitment, and thereafter phagocyte cellular debris of endothelial cells within the capillary lumen.

It is also probable that the beta-2 integrin CD11c must have a pivotal role for the recruitment and the activation of monocytes in AT during obesity,^27^ as in atherosclerotic plaque.^37^ Several study have already demonstrated that CD11c expression was increased on monocytes during obesity and hyperlipidemia in mice and in human.^37, 38^ More interestingly, it appears that CD11c increase specifically on ‘non-classical’ monocytes under an acute pro-inflammatory condition in insulin-resistant subjects (high-fat meal).^32^ All these studies are in favor of a crucial role of the subset of GR1^low^ monocytes in mice (or ‘non-classical’ monocytes in human) in metabolic diseases (obesity, hyperlipidemia and atherosclerosis).

It is important to point out that CX3CR1 in BM chimeric experiments is also potentially expressed on other hematopoietic cells such as natural killer cells, dendritic cells or T cells.^14, 23, 24, 25, 26, 27, 28, 29, 30, 31, 32, 33, 34, 35, 36, 37, 38, 39^ However, we did not detect any significant changes in the number of these cells in blood (Supplementary Figure 5) or in AT (data not shown) between CX3CR1^−/−^ and control female mice. In addition, the transgene *hBcl2* under the CD11c promoter might also be expressed in other myeloid cells, including dendritic cells.^24^ Therefore, we cannot exclude that in these different mice models, other hematopoietic actors influence the outcomes observed under HFD. However, when we pooled mice models together, a strong and significant inverse correlation was found between the proportion of Gr1^low^ monocyte subset and IR strongly supporting an active role of the Gr1^low^ subset in the control of metabolic homeostasis. This conclusion is reinforced by the fact that no correlation was found with the Gr1^high^ subset and the IR.

Our results initially seems to contrast with the work by Morris *et al.*^30^ who did not observed changes on BW, insulin sensitivity and populations of ATM of CX3CR1^−/−^ mice compared to controls fed a HFD. However, Lee *et al.*^40^ recently reported opposite results to this study (similar to our results) with an impaired glucose tolerance in CX3CR1^−/−^ mice, but driven by a defect in β-pancreatic cell functions. However, both studies were carried out in males while our study was performed in females. Similarly to Morris’s work, we did not find any differences in the BW gain and the insulin sensitivity upon chronic exposure to HFD between CX3CR1^−/−^ males and controls (data not shown). Interestingly, other studies reported a gender-specific effect in female mice fed a HFD^41, 42^ and demonstrated that some sex-specific factors can be carried out by hematopoietic cells.^41^ Moreover, a recent study of mice lacking Gr1^low^ monocytes (Nur4a1^−/−^) reported that the obesity phenotype was present only in female but not in male mice.^34^

In the present study, it is interesting to note that the decreased BW in CX3CR1^−/−^ mice as well as in recipients of CX3CR1^−/−^ BM mice was observed lately (after 18 weeks of HFD). Similarly, when we evaluated IR (GTT and ITT) before 18 weeks of diet, we did not found differences between the two genotypes (data not shown). Recently, Strissel *et al.*^43^ demonstrated in a kinetic study that inflammation of AT was increasing during the first 16 weeks of HFD, and decreasing on later time points. Hence, it is tempting to speculate that Gr1^low^ monocytes would have a protective anti-inflammatory role in the AT at late time point of HFD, to resolve inflammation. This hypothesis would explain late onset of weight and IR phenotype in CX3CR1^−/−^ mice. In the same way, mice deficient for Nur4a1, which have a very low level of Gr1^low^ monocytes, display a threefold increase in atherosclerosis development, and the lack of Gr1^low^ monocytes exacerbate inflammation most likely by an inability to resolve inflammation.^44^ These results are in accordance with those of Auffray *et al.*^10^ who demonstrated that the specific subset of Gr1^low^ monocytes patrol the vasculature and participate in the resolution of inflammation.

Low-grade inflammation is a key event in the physiopathology associated with obesity. Understanding the relative role of the different monocyte subsets in the induction of this meta-inflammation is crucial to better understand the development of the metabolic alteration linked to obesity and IR. Our study brings to light the new therapeutic challenge that the selective modulation of one or the other monocyte population could represent.

## References

[bib1] Lumeng CN, Saltiel AR. Inflammatory links between obesity and metabolic disease. J Clin Invest 2011; 121: 2111–2117. 21633179 10.1172/JCI57132 PMC3104776

[bib2] Hotamisligil GS. Inflammation and metabolic disorders. Nature 2006; 444: 860–867. 17167474 10.1038/nature05485

[bib3] McNelis JC, Olefsky JM. Macrophages, immunity, and metabolic disease. Immunity 2014; 41: 36–48. 25035952 10.1016/j.immuni.2014.05.010

[bib4] Xu H, Barnes GT, Yang Q, Tan G, Yang D, Chou CJ et al. Chronic inflammation in fat plays a crucial role in the development of obesity-related insulin resistance. J Clin Invest 2003; 112: 1821–1830. 14679177 10.1172/JCI19451 PMC296998

[bib5] Shoelson SE, Herrero L, Naaz A. Obesity, inflammation, and insulin resistance. Gastroenterology 2007; 132: 2169–2180. 17498510 10.1053/j.gastro.2007.03.059

[bib6] Lumeng CN, Bodzin JL, Saltiel AR. Obesity induces a phenotypic switch in adipose tissue macrophage polarization. J Clin Invest 2007; 117: 175–184. 17200717 10.1172/JCI29881 PMC1716210

[bib7] Shi C, Pamer EG. Monocyte recruitment during infection and inflammation. Nat Rev Immunol 2011; 11: 762–774. 21984070 10.1038/nri3070 PMC3947780

[bib8] Geissmann F, Jung S, Littman DR. Blood monocytes consist of two principal subsets with distinct migratory properties. Immunity 2003; 19: 71–82. 12871640 10.1016/s1074-7613(03)00174-2

[bib9] Geissmann F, Manz MG, Jung S, Sieweke MH, Merad M, Ley K. Development of monocytes, macrophages, and dendritic cells. Science 2010; 327: 656–661. 20133564 10.1126/science.1178331 PMC2887389

[bib10] Auffray C, Fogg D, Garfa M, Elain G, Join-Lambert O, Kayal S et al. Monitoring of blood vessels and tissues by a population of monocytes with patrolling behavior. Science 2007; 317: 666–670. 17673663 10.1126/science.1142883

[bib11] Tacke F, Alvarez D, Kaplan TJ, Jakubzick C, Spanbroek R, Llodra J et al. Monocyte subsets differentially employ CCR2, CCR5, and CX3CR1 to accumulate within atherosclerotic plaques. J Clin Invest 2007; 117: 185–194. 17200718 10.1172/JCI28549 PMC1716202

[bib12] Nahrendorf M, Swirski FK, Aikawa E, Stangenberg L, Wurdinger T, Figueiredo JL et al. The healing myocardium sequentially mobilizes two monocyte subsets with divergent and complementary functions. J Exp Med 2007; 204: 3037–3047. 18025128 10.1084/jem.20070885 PMC2118517

[bib13] Dal-Secco D, Wang J, Zeng Z, Kolaczkowska E, Wong CH, Petri B et al. A dynamic spectrum of monocytes arising from the *in situ* reprogramming of CCR2+ monocytes at a site of sterile injury. J Exp Med 2015; 212: 447–456. 25800956 10.1084/jem.20141539 PMC4387291

[bib14] Hilgendorf I, Gerhardt LM, Tan TC, Winter C, Holderried TA, Chousterman BG et al. Ly-6Chigh monocytes depend on Nr4a1 to balance both inflammatory and reparative phases in the infarcted myocardium. Circ Res 2014; 114: 1611–1622. 24625784 10.1161/CIRCRESAHA.114.303204 PMC4017349

[bib15] Varga T, Mounier R, Gogolak P, Poliska S, Chazaud B, Nagy L. Tissue LyC6- macrophages are generated in the absence of circulating LyC6- monocytes and Nur77 in a model of muscle regeneration. J Immunol 2013; 191: 5695–5701. 24133167 10.4049/jimmunol.1301445

[bib16] Carlin LM, Stamatiades EG, Auffray C, Hanna RN, Glover L, Vizcay-Barrena G et al. Nr4a1-dependent Ly6C(low) monocytes monitor endothelial cells and orchestrate their disposal. Cell 2013; 153: 362–7518. 23582326 10.1016/j.cell.2013.03.010 PMC3898614

[bib17] Poitou C, Dalmas E, Renovato M, Benhamo V, Hajduch F, Abdennour M et al. CD14dimCD16+ and CD14+CD16+ monocytes in obesity and during weight loss: relationships with fat mass and subclinical atherosclerosis. Arterioscler Thromb Vasc Biol 2011; 31: 2322–2330. 21799175 10.1161/ATVBAHA.111.230979

[bib18] Rogacev KS, Ulrich C, Blomer L, Hornof F, Oster K, Ziegelin M et al. Monocyte heterogeneity in obesity and subclinical atherosclerosis. Eur Heart J 2010; 31: 369–376. 19687164 10.1093/eurheartj/ehp308

[bib19] Cros J, Cagnard N, Woollard K, Patey N, Zhang SY, Senechal B et al. Human CD14dim monocytes patrol and sense nucleic acids and viruses via TLR7 and TLR8 receptors. Immunity 2010; 33: 375–386. 20832340 10.1016/j.immuni.2010.08.012 PMC3063338

[bib20] Landsman L, Bar-On L, Zernecke A, Kim KW, Krauthgamer R, Shagdarsuren E et al. CX3CR1 is required for monocyte homeostasis and atherogenesis by promoting cell survival. Blood 2009; 113: 963–972. 18971423 10.1182/blood-2008-07-170787

[bib21] Combadiere C, Potteaux S, Rodero M, Simon T, Pezard A, Esposito B et al. Combined inhibition of CCL2, CX3CR1, and CCR5 abrogates Ly6C(hi) and Ly6C(lo) monocytosis and almost abolishes atherosclerosis in hypercholesterolemic mice. Circulation 2008; 117: 1649–1657. 18347211 10.1161/CIRCULATIONAHA.107.745091

[bib22] Gautier EL, Huby T, Saint-Charles F, Ouzilleau B, Chapman MJ, Lesnik P. Enhanced dendritic cell survival attenuates lipopolysaccharide-induced immunosuppression and increases resistance to lethal endotoxic shock. J Immunol 2008; 180: 6941–6.23. 18453615 10.4049/jimmunol.180.10.6941

[bib23] Jung S, Aliberti J, Graemmel P, Sunshine MJ, Kreutzberg GW, Sher A et al. Analysis of fractalkine receptor CX(3)CR1 function by targeted deletion and green fluorescent protein reporter gene insertion. Mol Cell Biol 2000; 20: 4106–4114. 10805752 10.1128/mcb.20.11.4106-4114.2000 PMC85780

[bib24] Gautier EL, Huby T, Saint-Charles F, Ouzilleau B, Pirault J, Deswaerte V et al. Conventional dendritic cells at the crossroads between immunity and cholesterol homeostasis in atherosclerosis. Circulation 2009; 119: 2367–2375. 19380622 10.1161/CIRCULATIONAHA.108.807537

[bib25] Shearn AI, Deswaerte V, Gautier EL, Saint-Charles F, Pirault J, Bouchareychas L et al. Bcl-x inactivation in macrophages accelerates progression of advanced atherosclerotic lesions in Apoe(-/-) mice. Arterioscler Thromb Vasc Biol 2012; 32: 1142–1149. 22383704 10.1161/ATVBAHA.111.239111

[bib26] Westcott DJ, Delproposto JB, Geletka LM, Wang T, Singer K, Saltiel AR et al. MGL1 promotes adipose tissue inflammation and insulin resistance by regulating 7/4hi monocytes in obesity. J Exp Med 2009; 206: 3143–3156. 19995956 10.1084/jem.20091333 PMC2806469

[bib27] Wu H, Perrard XD, Wang Q, Perrard JL, Polsani VR, Jones PH et al. CD11c expression in adipose tissue and blood and its role in diet-induced obesity. Arterioscler Thromb Vasc Biol 2010; 30: 186–192. 19910635 10.1161/ATVBAHA.109.198044 PMC2830649

[bib28] Lumeng CN, Deyoung SM, Bodzin JL, Saltiel AR. Increased inflammatory properties of adipose tissue macrophages recruited during diet-induced obesity. Diabetes 2007; 56: 16–23. 17192460 10.2337/db06-1076

[bib29] Shaul ME, Bennett G, Strissel KJ, Greenberg AS, Obin MS. Dynamic, M2-like remodeling phenotypes of CD11c+ adipose tissue macrophages during high-fat diet-induced obesity in mice. Diabetes 2010; 59: 1171–1181. 20185806 10.2337/db09-1402 PMC2857897

[bib30] Morris DL, Oatmen KE, Wang T, DelProposto JL, Lumeng CN. CX3CR1 deficiency does not influence trafficking of adipose tissue macrophages in mice with diet-induced obesity. Obesity (Silver Spring) 2012; 20: 1189–119934. 22252034 10.1038/oby.2012.7 PMC4006981

[bib31] Nagareddy PR, Kraakman M, Masters SL, Stirzaker RA, Gorman DJ, Grant RW et al. Adipose tissue macrophages promote myelopoiesis and monocytosis in obesity. Cell Metab 2014; 19: 821–835. 24807222 10.1016/j.cmet.2014.03.029 PMC4048939

[bib32] Khan IM, Pokharel Y, Dadu RT, Lewis DE, Hoogeveen RC, Wu H et al. Postprandial monocyte activation in individuals with metabolic syndrome. J Clin Endocrinol Metab 2016; 101: 4195–4204. 27575945 10.1210/jc.2016-2732 PMC5095236

[bib33] Hanna RN, Carlin LM, Hubbeling HG, Nackiewicz D, Green AM, Punt JA et al. The transcription factor NR4A1 (Nur77) controls bone marrow differentiation and the survival of Ly6C- monocytes. Nat Immunol 2011; 12: 778–785. 21725321 10.1038/ni.2063 PMC3324395

[bib34] Perez-Sieira S, Martinez G, Porteiro B, Lopez M, Vidal A, Nogueiras R et al. Female Nur77-deficient mice show increase susceptibility to diet-induced obesity. PLoS ONE 2013; 8: e53836. 23342015 10.1371/journal.pone.0053836 PMC3544711

[bib35] Chao LC, Wroblewski K, Zhang Z, Pei L, Vergnes L, Ilkayeva OR et al. Insulin resistance and altered systemic glucose metabolism in mice lacking Nur77. Diabetes 2009; 58: 2788–2796. 19741162 10.2337/db09-0763 PMC2780886

[bib36] Satoh T, Kidoya H, Naito H, Yamamoto M, Takemura N, Nakagawa K et al. Critical role of Trib1 in differentiation of tissue-resident M2-like macrophages. Nature 2013; 495: 524–528. 23515163 10.1038/nature11930

[bib37] Wu H, Gower RM, Wang H, Perrard XY, Ma R, Bullard DC et al. Functional role of CD11c+ monocytes in atherogenesis associated with hypercholesterolemia. Circulation 2009; 119: 2708–2717. 19433759 10.1161/CIRCULATIONAHA.108.823740 PMC2716173

[bib38] Gower RM, Wu H, Foster GA, Devaraj S, Jialal I, Ballantyne CM et al. CD11c/CD18 expression is upregulated on blood monocytes during hypertriglyceridemia and enhances adhesion to vascular cell adhesion molecule-1. Arterioscler Thromb Vasc Biol 2011; 31: 160–166. 21030716 10.1161/ATVBAHA.110.215434 PMC3038249

[bib39] Combadiere C, Salzwedel K, Smith ED, Tiffany HL, Berger EA, Murphy PM. Identification of CX3CR1. A chemotactic receptor for the human CX3C chemokine fractalkine and a fusion coreceptor for HIV-1. J Biol Chem 1998; 273: 23799–23804. 9726990 10.1074/jbc.273.37.23799

[bib40] Lee YS, Morinaga H, Kim JJ, Lagakos W, Taylor S, Keshwani M et al. The fractalkine/CX3CR1 system regulates β cell function and insulin secretion. Cell 2013; 153: 413–425. 23582329 10.1016/j.cell.2013.03.001 PMC3717389

[bib41] Coenen KR, Gruen ML, Lee-Young RS, Puglisi MJ, Wasserman DH, Hasty AH. Impact of macrophage toll-like receptor 4 deficiency on macrophage infiltration into adipose tissue and the artery wall in mice. Diabetologia 2009; 52: 318–328. 19052722 10.1007/s00125-008-1221-7 PMC2615827

[bib42] Shi H, Kokoeva MV, Inouye K, Tzameli I, Yin H, Flier JS. TLR4 links innate immunity and fatty acid-induced insulin resistance. J Clin Invest 2006; 116: 3015–3025. 17053832 10.1172/JCI28898 PMC1616196

[bib43] Strissel KJ, Stancheva Z, Miyoshi H, Perfield JW, DeFuria J, Jick Z et al. Adipocyte death, adipose tissue remodeling, and obesity complications. Diabetes 2007; 56: 2910–2918. 17848624 10.2337/db07-0767

[bib44] Hanna RN, Shaked I, Hubbeling HG, Punt JA, Wu R, Herrley E et al. NR4A1 (Nur77) deletion polarizes macrophages toward an inflammatory phenotype and increases atherosclerosis. Circ Res 2012; 110: 416–427. 22194622 10.1161/CIRCRESAHA.111.253377 PMC3309661

